# A Randomized Controlled Trial on the Effect of Needle Gauge on the Pain and Anxiety Experienced during Radial Arterial Puncture

**DOI:** 10.1371/journal.pone.0139432

**Published:** 2015-09-25

**Authors:** Maxime Patout, Bouchra Lamia, Elodie Lhuillier, Luis-Carlos Molano, Catherine Viacroze, Daniel Benhamou, Jean-François Muir, Antoine Cuvelier

**Affiliations:** 1 Intensive Care Unit, Respiratory Department, Rouen University Hospital, Rouen, France; 2 Groupe de Recherche sur le Handicap Ventilatoire, UPRES EA 3830, Haute-Normandie Institute of Biomedical Research and Innovation, Rouen University, Rouen, France; Kurume University School of Medicine, JAPAN

## Abstract

**Background:**

Arterial punctures for assessment of arterial blood-gases can be a painful procedure. Lidocaine can be used to reduce pain prior to needle insertion but it is not a widely accepted practice. The purpose of this study was to determine whether a large size needle induces more pain compared to a smaller size needle for radial arterial puncture and to assess the anxiety associated with radial arterial punctures.

**Methods:**

We conducted a prospective, double-blind, randomized, controlled, monocentric study including all outpatients who had a planned assessment of arterial blood gas analysis. Patients were randomized to have the arterial puncture performed with a 23 or a 25 G needle. The main judgement criteria was pain during arterial puncture. Visual analogue scale for pain (VAS-P) and visual analogue scale for anxiety (VAS-A) were used to assess pain and anxiety during radial arterial puncture.

**Results:**

Two hundred consecutive patients were randomized. The 25 G needle was as painful as the 23 G needle (6.63 mm [0–19 mm] vs. 5.21 mm [0–18.49 mm], respectively, p = 0.527). Time for arterial puncture was longer with the 25 G needle than with the 23 G needle (42 s [35–55 s] vs. 33 s [24.5–35 s], respectively, p = 0.002). There was a correlation between the level of anxiety prior to the arterial puncture and the pain experienced by the patients (*p*: 0.369, p<0.0001). There was a correlation between the pain experienced by patients and the anxiety experienced in anticipation of another arterial puncture (*p*: 0.5124, p<0.0001).

**Conclusions:**

The use of 23 G needle allows quicker arterial sampling and is not associated with increased pain and symptoms. Anxiety was correlated with the pain experienced by patients during arterial punctures.

**Trial Registration:**

Clinicaltrials.gov: NCT02320916

## Introduction

Arterial blood-gas (ABG) measurements are the gold standard to evaluate pulmonary gas exchange [1]. However, arterial punctures are more painful than venous punctures [2] and, in ICU patients, cause greater anxiety than tracheal aspiration [3]. The only technique that has been shown to effectively reduce pain during arterial punctures is the subcutaneous injection of lidocaine [2,4]. However, this technique is more time consuming and is poorly used, even though it is recommended in Spanish guidelines [5–7] and teaching materials [8].

Capillary sampling of arterialised blood taken from the earlobe is another technique to assess pulmonary gas exchange. This technique is less painful than arterial puncture without subcutaneous anaesthesia [9]. Capillary sampling allows an adequate evaluation of pH and carbon-dioxide levels [10] but does not provide an accurate estimation of the level of oxygen [11,12]. Non-invasive technique such as transcutaneous oxymetry shares with capillary sampling its inaccuracy for the assessment of arterial oxygenation, especially when arterial oxygenation is low. Topical anaesthesia is widely used during arterial punctures despite the lack of proof of efficacy [13,14]. It is believed that the greater pain associated with arterial punctures is due to arterial innervation. It has also been shown that large gauge needles can cause more damage to the arterial wall [15]. As such, smaller gauge needles should cause less pain during arterial punctures. While performing arterial punctures with small gauge needles is feasible [16,17], to the best of our knowledge no studies have assessed the effect of needle gauge on arterial puncture related pain.

The aim of the present study was to compare the pain experienced during arterial punctures performed with a 25 G or 23 G needle. The secondary endpoints were the characterization of the pain and the anxiety associated with the arterial punctures.

## Materials and Methods

We conducted a monocentric, prospective, randomized, double-blind study.

### Patient Selection and Recruitment

All consecutive outpatients who had planned assessment of arterial blood gas as part of their routine clinical care in our Respiratory Department between April 11 and May 25, 2013, were asked to participate in the trial. Written and oral information was provided to all the patients. They were then asked to provide written informed consent for their participation in the study. Exclusion criteria included patients under the age of 18, inability to provide consent, or the presence of a contraindication to arterial punctures based on the American Association of Respiratory Care Guidelines [1].

### Ethics Statement

Our Institutional Review Board at Rouen University Hospital approved this study.

### Study Design

After inclusion, the patients were randomized based on instructions in sealed envelopes, which were prepared using a computer-generated randomization list by a third-party not involved in the randomization. A 23 G or a 25 G needle was assigned to each patient.

All arterial punctures were performed by one of the five nurses in the ambulatory unit without subcutaneous injection of lidocaine nor topical anaesthesia. The nurses who performed the arterial punctures were board certified nurses with at least 3 years experience in working in the respiratory ward. They collected anthropometric data from the patients, including the diameter of the punctured wrist. They also assessed the quality of the radial pulse, which was classified as imperceptible, weak, normal, strong, or visible. They assessed anxiety prior to the arterial punctures using a visual analogue scale for anxiety (VAS-A), which consisted of a horizontal 100-mm-long line with “no anxiety” indicated at the far left and “the highest level of anxiety that you can imagine” at the far right. Patients filled the VAS-A scale by themselves. Subjective quantification of this sort has been validated recently in recent studies on painful dental procedures [18]. In order to blind patients to the gauge of the needle, they were installed behind an opaque curtain through which they inserted their arm so that the nurse could perform the arterial puncture S1 Image. The duration of the arterial puncture was measured from needle insertion to full syringe filling.

During the medical consultation after the arterial puncture, the physician in charge of the patient, who was blinded to the needle gauge, assessed the pain felt during the arterial puncture using a visual analogue scale for pain (VAS-P), which was a horizontal 100-mm-long line with at the far left, “no pain” and “the highest level of pain that you can imagine” at the far right. Patients filled the VAS-P scale by themselves. If patients had multiple punctures, they were asked to rate their pain level for all the punctures performed. The patients were then asked about the most painful time of the procedure: needle insertion, during puncture, after puncture, repeat puncture, or no pain at all. They were also asked about their medical history, including tobacco use, risk factors for atheroma, and main respiratory diagnosis and whether they had undergone an arterial puncture in the past. The physician assessed the anxiety that patients may feel with respect to future arterial punctures using the same VAS-A described above.

Patients were followed-up until the end of the consultation with the physician. Eleven physicians were involved in the study.

The primary outcome was pain experienced during arterial puncture using VAS-P. Secondary outcomes were the anxiety experienced before and after arterial puncture using VAS-A, time to perform arterial puncture, the most painful moment of the procedure.

The present study was conducted in accordance with the amended Declaration of Helsinki. Our Institutional Review Board approved the protocol (CPP-SC 2011/01), and written informed consent was obtained from all patients prior the study. The study was registered with ANSM (Agence nationale de sécurité du médicament et des produits de santé/French Drug and Healthcare Product Safety Agency) (number 2010-A01373-36) prior its initiation. The study was registered at ClinicalTrials.gov (identifier NCT02320916) after completion, as it was already registered in the national registry. All the data were collected in Rouen Respiratory Department.

### Statistical Analysis

In order to detect a clinically significant 13-mm change in the pain score [19] with an alpha risk set at 0.05, a beta-risk set at 0.1 resulting in a power of 0.9 and an expected standard derivation set at 28mm based on the results of prior studies [2,13,14], a total of 196 patients were needed. We planned to include 200 patients in the study to ensure that we would have clinically significant results in the event of patient non-compliance or withdrawal.

Results are expressed as headcounts and percentages, means and standard deviation (SD) or medians, and first and third quartiles (IQR). Comparisons were performed using the Mann-Whitney, Wilcoxon match-paired ranking test, Kruskall-Wallis, ANOVA, and chi-square tests. Correlations were assessed using the Spearman correlation coefficient. A multiple linear regression was performed to look for independent variables related to the pain experienced by the patient. Following univariate analysis, a p value lower than 0.20 for a clinically relevant variable was necessary to enter stepwise multiple regression analysis. Assumptions of linearity and normality and no multicollinearity have been fulfilled before performing multiple regression analysis. Multiple linear regression was used for multivariate analysis. All tests were two-sided, the type I error rate was set at 0.05. The analyses were performed using GraphPad Prism 6^®^ for Mac OS X^®^ (GraphPad Software, La Jolla, CA, USA).

## Results

**Fig 1:**
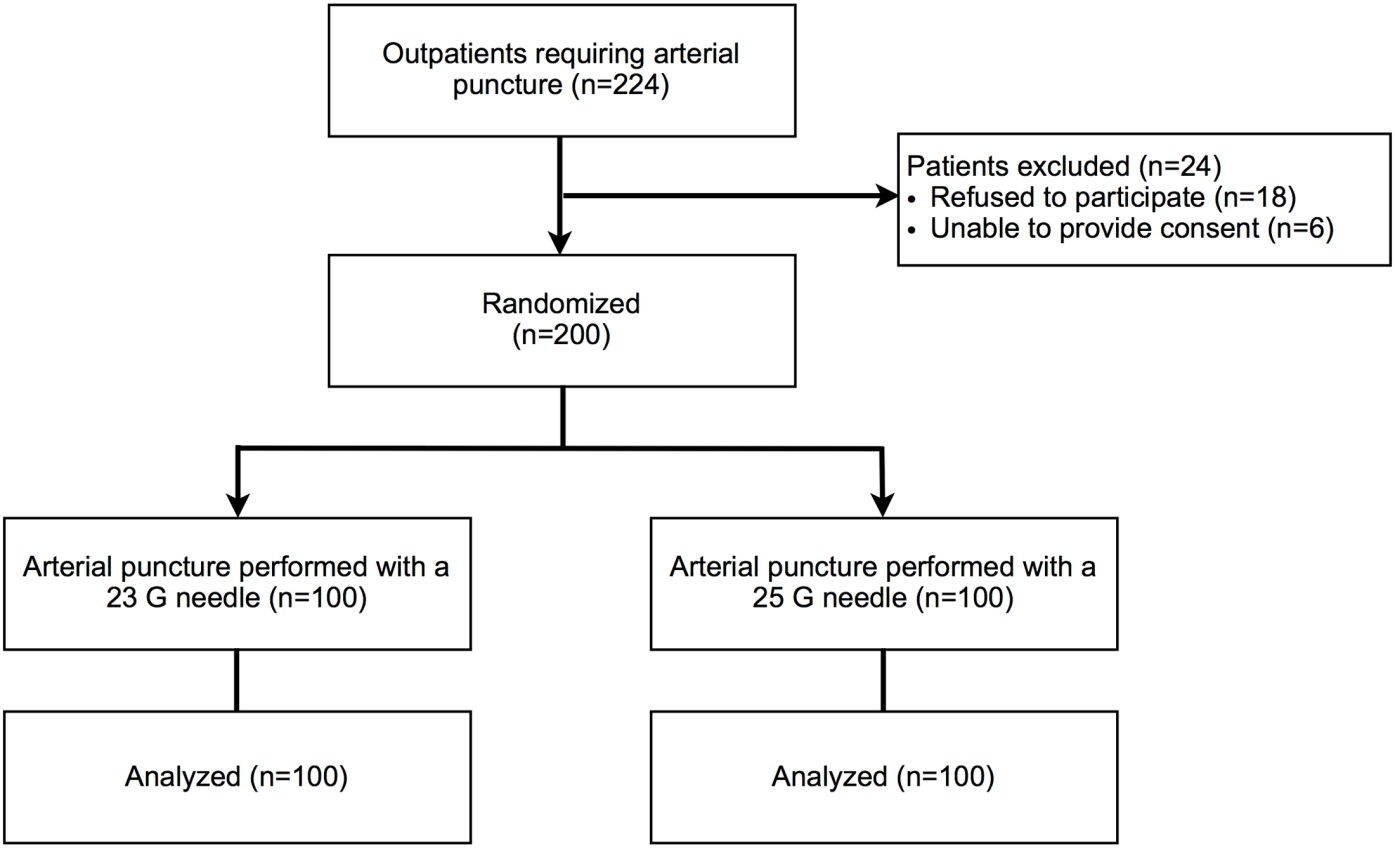
Flowchart.

Two-hundred-twenty-four consecutive patients were screened, and 200 were included in the study (Fig 1) between April 11 and May 25, 2013 after full recruitment was achieved. Of the 24 patients excluded from the study, 18 refused to give consent and 6 were unable to give consent. As shown in Table 1, there was no statistically significant difference between groups at baseline. The most common underlying respiratory disease was COPD (29% in the 23 G needle group, 30% in the 25 G needle group), and most of the patients had a history of tobacco use (62% in both group). At inclusion, most of the patients had undergone a previous arterial puncture (79% in the 23 G needle group, 78% in the 25 G needle group). Five experienced nurses performed a total of 210 arterial punctures. There was no statistical difference in the number of arterial punctures they performed in each group (p = 0.47).

**Table 1:** Characteristics of patients.

Variables	23 G needle (n = 100)	25 G needle (n = 100)	p
Age, mean (SD)	66.01 (13.17)	63.39 (15.21)	0.35
Gender, n(%)	53 (53%)	53 (53%)	1
Body Mass Index, mean (SD)	32.03 (8.24)	31.6 (9.42)	0.35
Underlying respiratory disease			
COPD, n(%)	29 (29%)	30 (30%)	
Asthma, n(%)	12 (12%)	6 (6%)	
OHS, n(%)	7 (7%)	6 (6%)	0.78
OSA, n(%)	24 (24%)	23 (23%)	
Other, n(%)	28 (28%)	35 (35%)	
Tobacco use			
Never smoked, n(%)	38 (38%)	38 (38%)	
Former smoker, n(%)	51 (51%)	46 (46%)	0.65
Active smoker, n(%)	11 (11%)	16 (16%)	
No previous arterial puncture, n(%)	21 (21%)	22 (22%)	0.86
Wrist diameter (cm), mean (SD)	17.84 (2.54)	17.68 (2.45)	0.42
Quality of radial pulse			
Pulse not felt, n(%)	2 (2%)	0 (0%)	
Weak pulse, n(%)	25 (25%)	34 (34%)	
Normal pulse, n(%)	56 (56%)	46 (46%)	0.13
Hard beating pulse, n(%)	16 (16%)	15 (15%)	
Visible pulse, n(%)	1 (1%)	5 (5%)	

**Fig 2:**
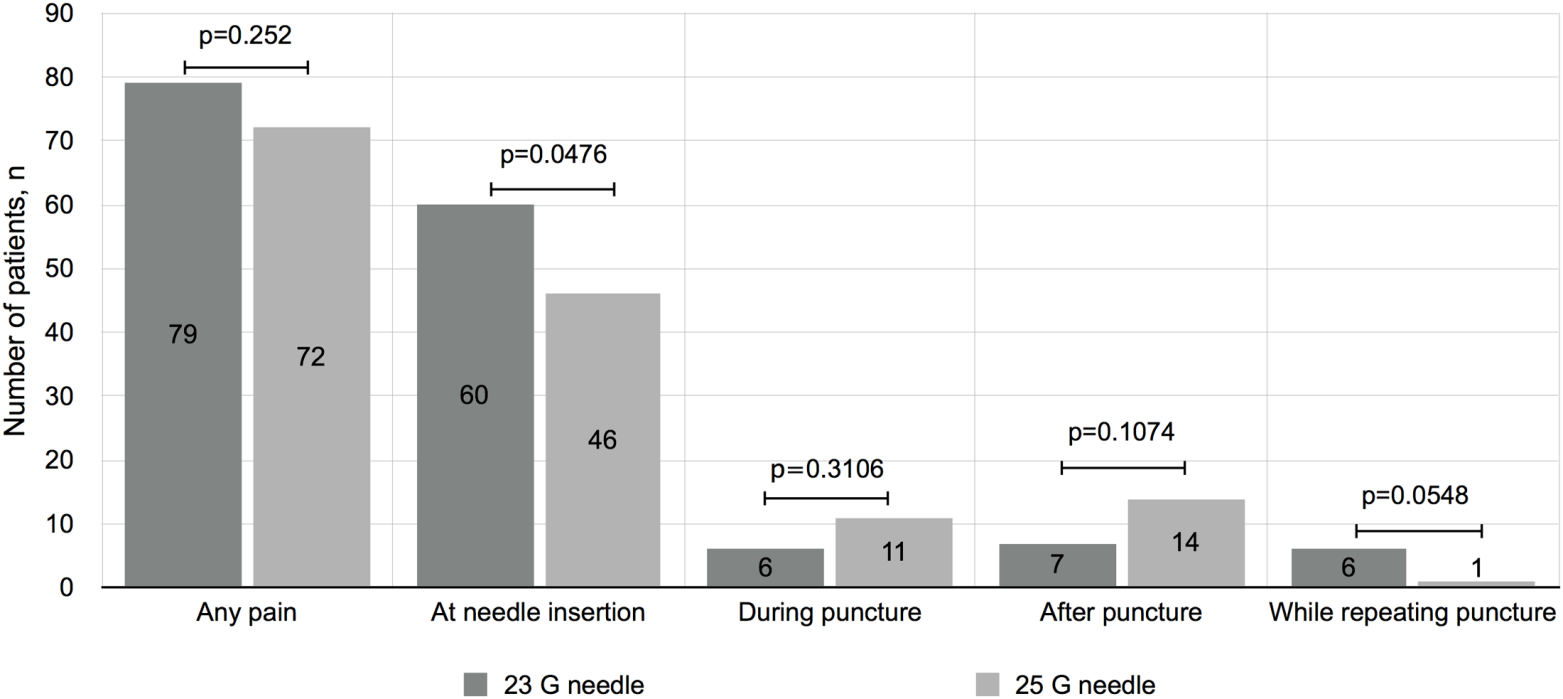
Most painful moment during arterial puncture. Distribution of the most painful time during the arterial punctures for each needle size (p = 0.0348).

There was no statistically significant difference in the pain experienced by patients using a 23 G needle (6.63 mm [0–19 mm]) or a 25 G needle (5.21 mm [0–18.49 mm]) (p = 0.53) (Table 2). There was no nurse-related difference in pain experienced by the patients (p = 0.49). There was a significant inter-group difference in terms of the most painful time during the arterial punctures (p = 0.03). In the 23 G needle group, pain was more frequent at needle insertion than in the 25 G needle group (p = 0.047) (Fig 2). Rate of failure to perform arterial puncture was similar in both group: 7% in the 23 G needle and 3% in the 25G needle (p:0.19). None of the patients had more than two arterial punctures in either arm. Seven of these ten patients reported that the repetition of the arterial punctures was the most painful experience. All second arterial punctures were successfully performed using the same needle.

**Table 2:** Time required for and pain and anxiety related to arterial punctures.

	23 gauge needle (n = 100)	25 gauge needle (n = 100)	p
Pain (mm), median [IQR]	6.63 [0–19]	5.21 [0–18.49]	0.53
Time required for AP (s), median [IQR]	33 [24.5–35]	42 [35–55]	0.002
Anxiety prior to AP (mm), median [IQR]	5.21 [0–27.9]	5.21 [0–24.7]	0.88
Anxiety after AP (mm), median [IQR]	1 [0–8]	1 [0–8]	0.99

Arterial punctures took significantly more time when using the 25 G needle (42 s [35–55 s]) compared to the 23 G needle (33 s [24.5–35 s]) (p = 0.002) (Table 2). There was no correlation between the time required for the arterial punctures and the pain experienced by the patients (p = 0.42) or between the time required for the arterial punctures and wrist diameters (p = 0.95). However, the correlation between the time required for the arterial punctures and the quality of the pulse was statistically significant. The arterial punctures took significantly more time for patients with the weakest pulses (*p*: -0.244, p<0.001).

**Fig 3:**
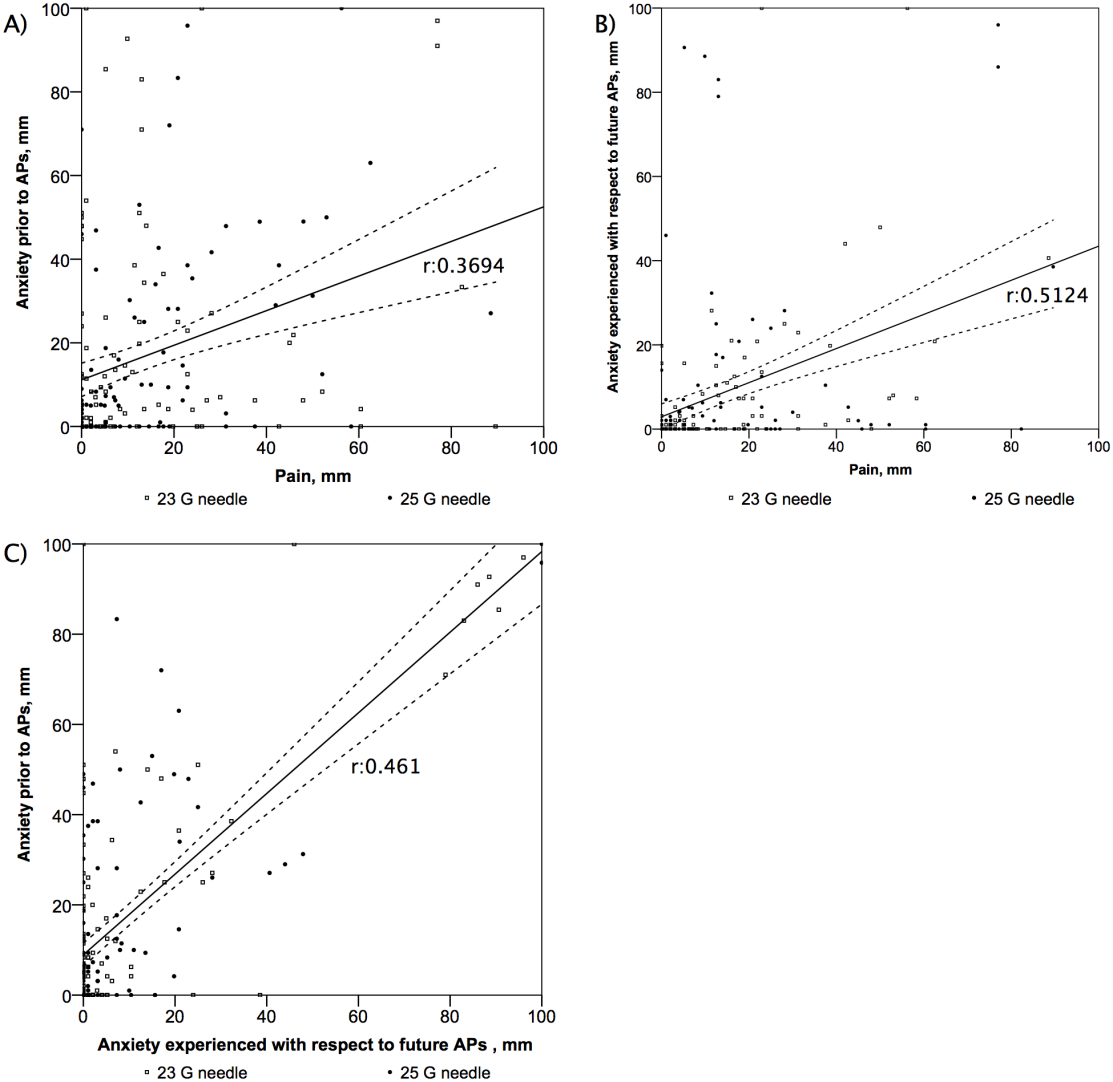
Correlations between anxiety and pain. (A) Correlation between anxiety prior to the arterial punctures and pain (rho:0.3694, p<0.001), (B) Correlation between the pain experienced during the arterial punctures and the anxiety experienced with respect to future arterial punctures (rho:0.5124, p<0.001), (C) Correlation between the anxiety experienced prior to the arterial punctures and the anxiety experienced with respect to future arterial punctures (rho:0.461, p<0.001).

The anxiety experienced by the patients in each group was similar prior to and after the arterial punctures (p = 0.88 and p = 0.99, respectively) (Table 2). In the overall study population, the anxiety experienced by the patients prior to the arterial punctures was moderately correlated with the pain experienced by the patients (*p*: 0.369, p<0.001). In the overall study population, the anxiety experienced by the patients with respect to future arterial punctures was correlated with the pain experienced during the arterial punctures (*p*: 0.512, p<0.001). In the overall study population, there was a correlation between the anxiety experienced with respect to future arterial punctures and the level of anxiety experienced prior to the arterial punctures (*p*: 0.461, p<0.001) (Fig 3). In the overall population multivariate analysis indicated that pain was related only to anxiety prior to arterial punctures (multiple r^2^ = 0.32, p <0.001) (Table 3). While there was a significant difference among nurses with respect to the level of anxiety experienced by the patients prior to the arterial punctures (p = 0.004), there was no difference with respect to the level of anxiety following the arterial punctures (p = 0.59). There was a decrease in the level of anxiety before and after the arterial punctures (5.28 mm [0–25 mm] and 1 mm [0–7.3 mm], respectively, p<0.001).

**Table 3:** Multivariate analysis: independent pain-related variables.

Variable	Correlation coefficient	Multiple correlation coefficient	Coefficient	Standard error	p
Anxiety prior to AP		0.31	0.24	0.05	<0.001
Time required for AP	0.09	-	-	-	NS
Numbers of previous AP	0.04	-	-	-	NS

NS = non significant

## Discussion

Our study showed that needle gauge has no significant impact on the pain experienced by patients during arterial punctures and that the use of small gauge needles lengthens the time required for the arterial punctures by about 10 s. Our study also provided more detailed informations on the type of pain experienced by patients and its correlation with anxiety.

Arterial blood pressure is correlated with the filling time of syringes with 23 G needles [20]. While we did not record the arterial blood pressure of our patients, the time required to fill the syringes may explain the lack of a significant difference in pain experienced by patients between the needle gauges. Patients in the 25 G group reported experiencing pain during the arterial punctures more frequently than patients in the 23 G group. This might be due greater nociceptive stimulation caused by the longer time required for needle insertion. On the other hand, the 23 G needle caused more pain at insertion, which may be due to the wider skin breach. Since the time required for arterial punctures was longer for patients in the 25 G group and since the level of pain experienced was similar to the 23 G group, we do not recommend the use of 25 G needles for arterial punctures.

Like previous interventional studies on pain associated to ABG, our study did not find any difference in the level of pain between each group. Interestingly, the level of pain experienced in our study is lower than the level of pain described in the literature. Indeed, the level of pain assessed was 6.63 and 5.21mm in each group. This level of pain is lower than the ones that have been reported in previous works in the placebo groups: 30.1 [2], 20.7 [13] and 23.8 [14]. We hypothesize that this difference may be due to the fact that trained nurses performed the arterial punctures. Recently, the effect of cryoanalgesia prior to arterial puncture was assessed and showed a significant reduction in the level of pain due to arterial puncture [21]. This study on cryoanalgesia was not double-blind but all the arterial puncture were performed by a same experimented pulmonary function technologist. In the intervention arm, the median level of pain was similar to the ones observed in our study.

To the best of our knowledge, our study is the first to assess the impact of anxiety on the pain experienced by patients during arterial punctures. Several studies have reported a correlation between anxiety and pain in other settings [22,23]. Our results showed that there is a correlation between the level of anxiety and the pain experienced by patients. This correlation is mild but was still significantly associated with pain in multiple linear regression. Even if the correlation between pain and anxiety is mild, reassurance of patient prior to puncture appears as a simple, non-pharmalogical and inexpensive way to decrease the level of pain felt by the patients. Thus, we think that providing explanations to patients and reassuring them may decrease the level of arterial puncture-related pain. This hypothesis is reinforced by our results showing that the level of anxiety reported by patients was significantly different with respect to the nurse performing the arterial puncture. This may be due to differences in the behaviour of the nurses. While the use of a VAS-A has not been validated to the same extent as VAS-P, previous studies have shown that a VAS-A is a reliable way to evaluate anxiety [18]. To maintain the blindness of our study, we prevented patients from seeing their wrist during the arterial punctures. We could not determine whether this had a positive or negative impact on the level of anxiety.

As pain management is now a cornerstone of the care that has to be provided to patients and as pulmonary gas exchange assessment remains central to manage patients with breathing disorder, we think further research should be led in that field. As they often tend to have repeated ABGs, patients in acute respiratory failure are of special interest. Indeed, rather than a precise isolated value, the kinetic of their gas exchange is a more relevant information as it guides physicians’ treatment. In that perspective, non-invasive tool such as transcutaneous capnography or electrical impedance tomography could be of particular interest.

Our study had several limitations. First, it was conducted in a single center with nurses experienced in performing arterial punctures and with patients in a steady state. We cannot thus extrapolate our results to patients with acute respiratory failure and to patients in whom arterial punctures would not be performed by experienced nurses. However, our study reproduced the daily clinical practice of chest physicians. Second, most of the patients had already experienced arterial punctures in the past. This may explain why the level of pain observed in our study was lower than in previous studies.

## Conclusion

Our study showed that pain associated with arterial puncture is not correlated with needle gauge and that anxiety is correlated with pain. We recommend the use of 23 gauge needles since they require less time than 25 gauge needles to perform the procedure. We also recommend that patients should be reassured prior to the arterial puncture. Further studies should be conducted to identify novel strategies to reduce the level of pain experienced by patients undergoing arterial punctures.

## Supporting Information

S1 File Research protocol. (PDF)

S2 File Dataset. (PDF)

S3 File Consort check list. (PDF)

S1 Image Setup to ensure that the patient is blind for the needle used by the nurse. Patients were installed on the right of the curtain and inserted their wrist between the curtains. Nurses were installed on the left of the curtain and performed the arterial puncture as well as the record the duration of the sampling using the chronometer.
(TIFF)
