# Supplementary material for: A Randomized Controlled Trial on the Effect of Needle Gauge on the Pain and Anxiety Experienced during Radial Arterial Puncture
Source: PLoS One. 2015 Sep 25;10(9):e0139432. doi: 10.1371/journal.pone.0139432 (PMC4583403; doi:10.1371/journal.pone.0139432)
Supplement: S1 File — (PDF) [file pone.0139432.s001.pdf]

Impact of the needle size in the pain experienced during arterial puncture
Comparative study of different needle size

Research in day-to-day practice
Registered to AFSSAPS with reference number: 2010-1-A01373-36
Submit to the Person's protection committee North-West I
Study administrator: Pneumology ward of Professor J-F Muir
Investigators: Dr Lamia / Patout

1. Rational:

Arterial blood sampling is essential to provide appropriate care to patients with acute or chronic disorder. The arterial blood sampling is usually repeated overtime for the follow-up of the disease. The arterial blood sampling provides greater pain than venous puncture. This might be due to the innervation of the arterial wall and by the fact that arteries are located deeper than veins [1]. For the ICU inpatients, arterial blood gas sampling cause greater anxiety than tracheal suctions [2].

A few methods have been proposed to reduce the pain associated with arterial blood gas sampling:

- Use of topical anaesthetic (such as EMLA®): Two randomized controlled studies against placebo have been [3,4]. None of them proved any efficacy with respect to lowering pain. Furthermore these topical anaesthetics have to be applied at least 60 minutes prior to the arterial puncture. We conducted an interview of 28 carers from the ward of Pneumology and 24 of them said that the delay of 60 minutes held them back from using these topical anaesthetic.
- Use of a subcutaneous injection of xylocaïne prior to puncture: A study has shown that this technique was efficient to reduce pain [5]. Yet, this technique is not used. [6]. In our hospital, out of the 28 persons interviewed, only one had used this technique at the beginning of his training but hasn't used it since then.

The use of small size needle (25Gauge) to perform arterial puncture is common. Its use for 11 5000 consecutive samplings have been reported with no noticeable side-effect [7, 8]. The use of small needle is up to the assessment and habits of the carers that perform the arterial puncture. To this day, no study has evaluated the use of small needle to reduce the pain experienced by patients during arterial puncture.

Impact of the needle size in the pain experienced during arterial puncture
Comparative study of different needle size

Research in day-to-day practice
Registered to AFSSAPS with reference number: 2010-1-A01373-36
Submit to the Person's protection committee North-West I
Study administrator: Pneumology ward of Professor J-F Muir
Investigators: Dr Lamia / Patout

2. Aim of the study:

The aim of the study is:

1- To compare the level of pain assessed by a visual analogue scale (VAS) experienced by the patient during arterial puncture performed with a 25 or with a 23 Gauge needle.

3. Patients et methods:

The study is monocentric and prospective.

It will be held in the respiratory outpatient clinic of Pr. Jean-François Muir, Bois-Guillaume Hospital, University Hospital of Rouen from May to June 2011.

The investigators of the study will be Dr. Bouchra Lamia and Maxime Patout (Fellow in respiratory medicine).

The promoter of the study will be the Intensive care respiratory unit & Pneumology ward of Pr. Jean-François Muir, Bois-Guillaume Hospital, University Hospital of Rouen. Both promoter and investigators states that the study will be conducted with respect to the protocol and current legislation and regulations.

- **Inclusion criteria**

All the outpatients referred for a consultation with a chest physician working in the Respiratory Ward of Pr. J-F Muir with arterial blood puncture will be eligible for the study. The entire arterial puncture will have to be planned before the consultation and be a part of usual care of the patient.

- **Exclusion criteria**

Patients will be excluded from the study if: Age under 18 years, civil unable, imprisoned, they have applied topical anaesthetic before puncture, they request a specific needle size or another sampling methods (capillary sampling).

Impact of the needle size in the pain experienced during arterial puncture
Comparative study of different needle size

Research in day-to-day practice
Registered to AFSSAPS with reference number: 2010-1-A01373-36
Submit to the Person's protection committee North-West I
Study administrator: Pneumology ward of Professor J-F Muir
Investigators: Dr Lamia / Patout

- **Study protocol**

Screening and inclusion: With respect to inclusion and exclusions criteria, outpatients will receive the information booklet and then they'll be consecutively and prospectively included.

Information: At welcome desk of the outpatient clinic, patients will receive an information booklet explaining the aims and modalities of the study.

Randomization: Nurse will perform randomization to the 25 or to the 23 Gauge needle before preparing its material for sampling.

Blinding: Before performing the arterial puncture, an opaque curtain will we placed between the patient's wrists and its eyes so that he can't see the type of needle used by the nurse. The chest physician will assess pain experienced during puncture during its consultation. The chest physician will be blind of the needle that has been used for the patient.

Data collection: Data will be collected on a standardized anonym case record file.

Before performing arterial puncture, nurse will collect demographic and anthropometric data: age, sex, weight, height, wrist diameter and the quality of the radial pulse, which was classified as imperceptible, weak, normal, strong, or visible. Time to perform arterial puncture will be recorded by the nurse. The duration of the arterial puncture will be measured from needle insertion to full syringe filling. After sampling, the nurse will report the number of attempts, why he had to perform multiple puncture, if he failed to perform the puncture. Failure of the puncture will define by failure to perform arterial sampling after 3 tries. The reason of the failure will be reported.

After arterial sampling, the chest physician will assess the level of pain using a visual analogue scale. He will also assess the most painful time of the procedure: needle insertion, during puncture, after puncture, repeat puncture, or no pain at all. Finally, the chest physician will assess the cardiovascular risks factor and the number of arterial

puncture that the patient already had.

Duration of the study: Patients will be involved in the study from their inclusion to the end of the consultation with the chest physician. For each patient, the duration of its participation to the study will be significantly less than 6 hours. Data collection and arterial sampling won't take more than 10 minutes.

Patients' follow-up: No additional follow-up will be necessary. Patients' follow-up will be organized as decided by the chest physician.

Withdrawal of the study: Patients will be free to withdraw from the study at any time by simple oral notification of the nurse or the chest physician.

4. Statistical Analysis:

- **Methods**

Results are expressed as headcounts and percentages, means and standard deviation (SD) or medians, and first and third quartiles (IQR). Comparisons were performed using the Mann-Whitney, Wilcoxon, Kruskall-Wallis, ANOVA, and chi-square tests. All tests were two-sided, the type I error rate was set at 0.05 and the power was set at 90%. No intermediate analysis is planned.

- **Number of patients needed**

With regard to previous publication on pain, a clinically significant change in the pain corresponds to a change of 13-mm on a 100-mm visual analogue scale. With respect to previous published data on the subject using a similar methodology, the expected standard derivation was set at 28mm.
196 patients are needed with the alpha risk set at 0.05, the ß-risk set at 0.9 and with a bilateral formulation of the hypothesis. All the patients will we included in the outpatient clinic of the Respiratory Ward, Bois-Guillaume Hospital – University hospital of Rouen.

5. Expected results:

We are aim at finding a significant difference in the pain experienced by patients when arterial puncture was performed with a 25 Gauge needle as compared to those of which arterial puncture was performed with a 23 Gauge needle.

6. Right to access to data and documents:

The administrator: Intensive Care Respiratory Unit & Pneumology ward – EA3830 is the owner of the data. The will no be use or transmit to a third party without its approval.

7. Control and quality management:

Experienced nurses trained to perform arterial puncture will collect data. A chest physician will collect medical data.

8. Independent watching comity

There will be no independent watching comity

9. Ethical evaluation of the particular modalities of follow-up described in the protocol

There is no follow-up planned in the protocol.

10. Use of data and conservation of the documents and data linked to the research

Before study initiation, the use of the data will be validated by the Consultative Comity for the treatment of Information in the field of research in health (Comité Consultatif sur le Traitement de l'Information en matière de Recherche dans le domaine

de la Santé - CCTIRS) and b the National commission on liberty and informatics (Commission Nationale de l'Informatique et des Libertés - CNIL). The study will be declared by itself.

The patient information booklet will include all the information regarding to the protection of patients participating to research.

11. References

1 - Hudson et al. Use of local anaesthesia for arterial punctures. American journal of critical care : an official publication, American Association of Critical-Care Nurses (2006) vol. 15 (6) pp. 595-9

2 - Turner JS, Briggs SJ, Springhorn HE, Potgieter PD. Patients' recollection of intensive care unit experience. Cr Care Med (1990) vol. 18 pp.966-8.

3 - Tran et al. A randomized controlled trial of the effectiveness of topical amethocaine in reducing pain during arterial puncture. Chest (2002) vol. 122 (4) pp. 1357-60

4 - Aaron et al. Topical tetracaine prior to arterial puncture: a randomized, placebo-controlled clinical trial. Respiratory medicine (2003) vol. 97 (11) pp. 1195-9

5 - Giner et al. Pain during arterial puncture. Chest (1996) vol. 110 (6) pp. 1443-5

6 - Sado et Deakin. Local anaesthesia for venous cannulation and arterial blood gas sampling: are doctors using it?. Journal of the Royal Society of Medicine (2005) vol. 98 (4) pp. 158-60

7 - Brown et al. The reliability of the 25-gauge needle for arterial blood sampling. Anesthesiology (1972) vol. 37 (3) pp. 363

8 - Sabin et al. Clinical experience using a small-gauge needle for arterial puncture. Chest (1976) vol. 69 (3) pp. 437-9
