# Supplementary material for: A Randomized Controlled Trial on the Effect of Needle Gauge on the Pain and Anxiety Experienced during Radial Arterial Puncture
Source: PLoS One. 2015 Sep 25;10(9):e0139432. doi: 10.1371/journal.pone.0139432 (PMC4583403; doi:10.1371/journal.pone.0139432)
Supplement: S2 File — (PDF) [file pone.0139432.s002.pdf]

Number	Date	Nurse	Arm	First ABG	Weight	Height	BMI	Gender	Age	Right wrist diameter	left wrist diameter	Pulse quality	Duration ABG	Nb attemps	Reason of failure	Anxiety prior ABG	Pain after ABG	Anxiety after ABG	Médecin	Most painful moment	Nb of anterior ABG	Délaidernier GdS	Main respiratory disease	Smoking history	Pack-Year	Dyslipidemia	High blod pressure	Diabetes
1	11-avr-2013	1	2	0	187	1,72	63,2	1	61	21,5	NA	3	42	1	NA	1	2	3	DB	2	1	1	1	1	50	1	1	1
2	11-avr-2013	1	2	0	60	1,70	20,8	1	83	19	NA	2	33	1	NA	0	0	0	PP	4	1	0	1	1	70	0	0	0
3	11-avr-2013	1	1	0	96	1,65	35,3	1	76	NA	20	2	25	1	NA	5	8	0	FP	0	1	2	1	1	10	0	1	0
4	11-avr-2013	2	1	0	97	1,66	35,2	0	51	19	NA	2	19	1	NA	1	0	0	DB	0	1	0	5	1	NA	0	0	0
5	11-avr-2013	1	1	0	48	1,64	17,8	1	64	NA	16	2	22	1	NA	4	0	0	DP	4	1	0	5	1		0	1	0
6	11-avr-2013	5	1	0	104	1,57	42,2	0	70	20	NA	3	37	1	NA	53	13	15	CV	0	1	0	3	1	NA	1	1	0
7	11-avr-2013	2	2	0	55	1,60	21,5	0	77	15,7	NA	2	46	1	NA	13	11	0	DP	1	1	2	1	1		1	1	0
8	11-avr-2013	2	2	0	119	1,62	45,3	0	60	19,5	NA	1	28	1	NA	97	77	96	CV	1	1	2	3	1	0	1	1	1
9	11-avr-2013	1	2	0	74	1,64	27,5	0	64	NA	17,9	1	33	1	NA	0	4	0	DP	2	1	2	2	1	NA	0	0	0
10	11-avr-2013	1	1	0	112	1,80	34,6	1	68	NA	19	1	48	1	NA	5	5	0	CV	1	1	0	1	1		0	1	1
11	12-avr-2013	5	1	0	123	1,77	39,3	1	79	NA	21,3	2	27	1	NA	49	48	0	CV	0	1	0	3	1	NA	0	1	0
12	12-avr-2013	5	2	0	69	1,80	21,3	1	51	NA	17	1	40	1	NA	100	26	0	CV	0	1	0	1	1	30	0	0	0
13	12-avr-2013	5	2	0	82	1,61	31,6	0	74	NA	20	2	50	1	NA	91	77	86	DS	0	1	0	1	1	40	0	1	0
14	12-avr-2013	5	1	0	86	1,80	26,5	1	40	NA	19,5	2	32	1	NA	0	0	0	HA	4	1	2	5	0	17	1	0	1
15	12-avr-2013	4	1	0	80	1,81	24,4	1	75	20	NA	3	95	1	NA	10	13	8	LCMV	2	1	2	5	1	5	0	0	0
16	15-avr-2013	3	2	0	95	1,63	35,8	1	80	NA	18	3	30	1	NA	17	7	5	DS	0	1	0	4	1	60	1	1	0
17	16-avr-2013	3	2	0	85	1,80	26,2	1	53	17,5	17	2	68	2	0	20	45	2	LCMV	2	1	2	5	0	40	0	0	0
18	16-avr-2013	3	2	1	84	1,70	29,1	1	65	20	NA	2	30	1	NA	0	4	4	JFM	1	0	NA	3	1	5	1	1	0
19	16-avr-2013	3	2	0	95	1,62	36,2	0	58	17	0	1	54	1	NA	54	1	7	LCMV	4	1	0	5	1	NA	1	1	0
20	17-avr-2013	3	1	0	79	1,71	27,0	1	71	17,5	NA	2	120	1	NA	7	7	0	DB	2	1	0	1	1		0	1	0
21	17-avr-2013	3	2	0	51	1,72	17,2	1	78	NA	16	4	40	1	NA	100	1	46	DB	2	1	0	1	1	100	0	0	0
22	17-avr-2013	3	1	0	91	1,70	31,5	1	61	17,5	NA	2	100	1	NA	10	15	11	DB	0	1	1	1	1	45	0	0	0
23	17-avr-2013	2	1	0	63	1,76	20,3	1	75	17,5	NA	3	36	1	NA	72	19	17	DB	0	1	0	5	1	NA	0	1	0
24	17-avr-2013	2	1	1	99	1,82	29,9	1	56	19,3	NA	3	28	1	NA	0	2	0	DB	0	0	NA	1	1	80	0	0	0
25	18-avr-2013	3	2	0	89	1,68	31,5	1	81	18	NA	1	50	1	NA	0	0	0	FP	4	1	2	4	1	17	1	1	1
26	18-avr-2013	3	2	0	70	1,69	24,5	1	82	17	NA	1	60	1	NA	71	13	79	FP	0	1	0	5	1	NA	1	1	0
27	18-avr-2013	3	1	1	92	1,80	28,4	1	70	18	NA	2	120	1	NA	0	19	0	DB	1	0	NA	5	1	20	1	1	0
28	18-avr-2013	3	2	0	65	1,54	27,4	0	71	NA	15,5	1	48	1	NA	0	12	2	DP	0	1	0	1	1		0	0	0
29	18-avr-2013	5	2	0	94	1,70	32,5	1	59	20	NA	2	41	1	NA	12	0	0	DP	1	1	0	1	1		0	0	0
30	19-avr-2013	2	1	0	97	1,56	39,9	0	60	NA	18	2	52	1	NA	0	0	0	CV	0	1	0	4	1	NA	0	1	1
31	19-avr-2013	2	1	0	122	1,70	42,2	0	63	NA	20,5	2	33	1	NA	0	0	0	LCMV	4	1	0	2	1	6	0	1	0
32	19-avr-2013	5	1	0	95	1,82	28,7	1	69	NA	20,3	2	33	1	NA	50	53	8	HA	0	1	1	3	1		0	1	0
33	19-avr-2013	5	1	0	67	1,58	26,8	0	61	16	NA	3	25	1	NA	5	2	0	HA	0	1	2	3	1	NA	1	0	1
34	19-avr-2013	4	1	0	92	1,67	33,0	1	79	18	NA	3	14	1	NA	46	0	0	JFM	3	1	2	3	1	NA	1	1	1
35	19-avr-2013	5	2	0	92	1,64	34,2	0	47	NA	17	2	40	1	NA	83	13	83	HA	0	1	0	5	0	41	0	0	0
36	19-avr-2013	2	2	0	88	1,69	30,8	1	48	NA	17,7	3	34	1	NA	0	5	0	JFM	0	1	2	3	1	NA	0	1	0
37	19-avr-2013	2	1	0	78	1,60	30,5	1	83	16,8	NA	2	30	1	NA	0	0	0	LCMV	4	1	1	3	1	NA	0	0	0
38	19-avr-2013	5	1	0	85	1,62	32,4	1	98	0	20	4	15	1	NA	0	2	1	LCMV	4	1	2	1	1		0	0	0
39	19-avr-2013	5	2	1	80	1,60	31,3	0	60	16,5	NA	2	15	1	NA	50	0	14	HA	4	0	NA	3	1	NA	0	0	0
40	19-avr-2013	4	2	0	68	1,60	26,6	0	70	16,5	NA	3	58	1	NA	2	0	0	JFM	0	1	2	5	1	NA	1	0	0
41	19-avr-2013	2	1	1	108	1,61	41,7	0	64	NA	18,3	2	37	1	NA	16	8	0	JFM	0	0	NA	5	1	NA	0	0	0
42	19-avr-2013	4	2	0	83	1,63	31,2	0	89	16,5	NA	3	66	1	NA	7	3	0	JFM	0	1	2	5	1	NA	1	1	0
43	19-avr-2013	5	2	1	57	1,72	19,3	0	52	17,5	NA	2	35	1	NA	0	7	0	JFM	4	0	NA	2	1	NA	0	0	0
44	19-avr-2013	4	2	1	130	1,68	46,1	0	38	NA	18	2	50	1	NA	48	14	17	JFM	0	0	NA	5	0		0	0	0
45	19-avr-2013	5	1	0	57	1,56	23,4	0	68	15	NA	2	25	1	NA	0	5	1	JFM	0	1	2	2	1	NA	0	0	0
46	22-avr-2013	1	1	1	89	1,75	29,1	1	63	19	NA	1	110	2	0	34	16	21	PP	1	0	NA	1	0		0	0	0
47	22-avr-2013	4	2	0	58	1,50	25,8	0	92	15	NA	3	55	1	NA	7	30	4	PP	0	1	0	1	#NUL!		#NUL!	#NUL!	#NUL!
48	22-avr-2013	1	1	1	84	1,76	27,1	0	67	NA	18	3	40	1	NA	1	17	10	PP	0	0	NA	1	1	10	0	0	0
49	22-avr-2013	4	1	0	60	1,60	23,4	0	77	NA	15	3	49	1	NA	4	0	0	AC	4	1	0	5	1	NA	1	1	0
50	22-avr-2013	1	1	0	75	1,57	30,4	0	54	16	NA	1	34	1	NA	29	42	44	AC	0	1	0	1	0		0	0	0
51	23-avr-2013	1	2	0	132	1,52	57,1	0	61	23	NA	2	60	1	NA	4	24	0	FP	0	1	0	4	1	NA	1	1	1
52	23-avr-2013	1	1	0	95	1,58	38,1	0	82	NA	22	1	34	1	NA	0	1	0	LCMV	4	1	0	4	1	NA	0	1	0
53	23-avr-2013	4	2	0	82	1,83	24,5	1	71	17	NA	2	65	1	NA	12	5	7	BL	0	#NUL!	2	1	0	18	0	0	0
54	24-avr-2013	4	1	0	137	1,72	46,3	1	38	19	NA	2	47	1	NA	9	0	0	JFM	4	1	2	3	1	NA	1	0	1
55	24-avr-2013	4	2	0	70	1,68	24,8	0	76	NA	17	4	36	1	NA	2	2	0	JFM	0	1	2	1	1		1	1	0
56	24-avr-2013	3	2	1	62	1,60	24,2	0	56	NA	16	2	70	1	NA	0	0	0	JFM	4	0	NA	5	1	NA	1	1	0
57	24-avr-2013	4	1	0	76	1,69	26,6	1	62	18	NA	2	32	1	NA	5	0	0	JFM	0	1	0	1	1	50	0	0	0
58	24-avr-2013	3	1	1	139	1,63	52,3	0	60	NA	20,5	1	50	1	NA	0	2	1	JFM	0	0	NA	5	0		1	1	0
59	24-avr-2013	3	2	1	67	1,65	24,6	1	63	NA	17	2	50	1	NA	0	0	0	JFM	2	0	NA	3	1		1	1	0
60	24-avr-2013	4	1	0	102	1,63	38,4	0	59	NA	19	2	50	1	NA	71	0	#NUL!	JFM	2	0	NA	3	1		1	1	0
61	24-avr-2013	1	2	0	76	1,64	28,3	0	61	NA	19	1	70	1	NA	0	0	0	JFM	1	1	1	3	1	NA	0	1	0
62	24-avr-2013	3	2	0	85	1,71	29,1	1	77	NA	19	2	42	1	NA	27	0	0	JFM	4	1		3	1	NA	0	1	0
63	24-avr-2013	4	1	0	104	1,75	34,0	0	72	NA	19	2	48	1	NA	2	0	1	JFM	4	1	2	3	1	NA	0	1	0
64	24-avr-2013	3	2	0	92	1,75	30,0	1	69	19	NA	4	35	1	NA	8	2	1	JFM	0	1	0	5	1		0	0	0
65	24-avr-2013	4	1	0	154	1,67	55,2	0	33	17	NA	2	48	1	NA	6	7	1	JFM	2	1	0	3	1	NA	0	0	0
66	24-avr-2013	3	2	0	72	1,48	32,9	0	69	16	NA	2	42	1	NA	0	20	1	DB	0	1	0	1	0	50	0	1	0
67	24-avr-2013	4	1	1	104	1,53	44,4	0	34	NA	16,5	2	120	1	NA	0	3	0	JFM	1	0	NA	5	0		1	0	1
68	24-avr-2013	3	2	0	118	1,58	47,3	0	57	NA	18	1	55	1	NA	0	0	0	JFM	4	1		3	1		0	0	0
69	24-avr-2013	3	2	1	145	1,58	58,1	0	25	NA	20	1	37	1	NA	0	0	0	JFM	4	0	NA	5	1		0	1	0
70	24-avr-2013	4	1	0	52	1,59	20,6	0	69	NA	15,5	0	122	1	NA	5	0	0	DB	0	1	0	5	1	NA	1	0	1
71	24-avr-2013	3	2	0	103	1,52	44,6	0	40	17,5	NA	1	38	1	NA	51	0	0	JFM	4	1	2	5	1	NA	0	0	0
72	24-avr-2013	4	1	0	95	1,76	30,7	1	80	19	NA	3	20	1	NA	49	39	20	DB	0	1	1	1	1	NA	1	1	0
73	24-avr-2013	1	2	0	118	1,80	36,4	1	63	20	NA	2	58	1	NA	4	19	0	DB	1	1	0	1	1	25	0	1	1
74	24-avr-2013	4	2	0	77	1,64	28,6	1	73	18	NA	2	53	1	NA	5	0	0	JFM	4	1	2	5	1		0	0	0
75	24-avr-2013	4	2	0	83	1,72	28,1	1	52	17,5	NA	3	40	1	NA	4	60	1	JFM	1	1	2	3	0		0	0	0
76	24-avr-2013	3	1	0	93	1,90	25,8	1	73	19,5	NA	3	38	1	NA	0	0	0	JFM	4	1	2	2	1		1	1	0
77	24-avr-2013	3	1	0	72	1,63	27,1	0	76	NA	16,5	3	45	1	NA	0	2	1	JFM	4	1	1	5	1	NA	1	1	0
78	24-avr-2013	4	2	9	91	1,71	31,1	1	75	18	NA	3	45	1	NA	5	17	0	JFM	0	1	2	1	1	NA	1	1	0
79	##########	1	1	0	83	1,60	32,4	1	80	21	NA	2	38	1	NA	3	0	0	DB	4	1	1	1	1	10	0	1	0
80	24-avr-2013	4	2	0	78	1,69	27,3	1	67	NA	18	3	40	1	NA	1	5	0	JFM	4	1	2	3	1		1	0	0
81	24-avr-2013	1	2	1	117	1,64	43,5	0	32	NA	20,5	2	36	1	NA	14	7	0	JFM	0	0	NA	5	1	NA	0	0	0
82	24-avr-2013	3	2	1	89	1,78	28,1	1	67	18	NA	3	28	1	NA	0	6	5	DB	4	0	NA	1	1	80	0	0	0
83	24-avr-2013	1	1	1	75	1,68	26,6	0	61	16	NA	1	138	2	0	3	31	3	JFM	3	0	NA	5	1		1	0	0
84	24-avr-2013	4	1	1	115	1,65	42,2	0	44	NA	17	2	40	1	NA	0	17	0	JFM	2	0	NA	5	1		0	0	1
85	24-avr-2013	1	2	1	89	1,76	28,7	1	59	18,5	NA	1	47	1	NA	48	0	0	JFM	4	0	NA	1	0	NA	1	0	0
86	24-avr-2013	4	2	0	123	1,67	44,1	0	42	18	NA	3	20	1	NA	2	1	0	JFM	2	0	NA	5	1	NA	0	0	0
87	24-avr-2013	4	1	1	93	1,57	37,7	0	78	20	NA	2	29	1	NA	7	5	2	DB	2	0	NA	5	1	NA	0	1	1
88	24-avr-2013	4	1	1	110	1,69	38,5	0	43	18	NA	2	40	1	NA	5	4	3	JFM	0	0	NA	5	1		0	0	0
89	25-avr-2013	4	2	0	104	1,62	39,6	0	50	18	NA	2	48	1	NA	85	5	91	DB	1	1	1	4	0	5	0	0	0
90	25-avr-2013	1	1	1	94	1,72	31,8	1	79	NA	19	2	16	1	NA	3	0	3	DB	0	1	1	1	1	10	0	1	0
91	25-avr-2013	1	2	0	#NUL!	#NUL!	#NUL!	1	51	NA	15	1	140	1	NA	23	23	13	PP	1	1	0	1	0	60	0	0	0
92	25-avr-2013	1	1	0	83	1,56	34,1	0	77	17	NA	2	28	1	NA	0	18	0	DB	0	1	1	5	1	NA	1	1	1
93	25-avr-2013	1	1	0	#NUL!	#NUL!	#NUL!	1	54	NA	16	#NUL!	160	1	NA	43	17	13	PP	0	1	0	1	0	35	0	1	0
94	25-avr-2013	4	2	0	59	1,63	22,2	1	82	17	NA	2	60	1	NA	0	0	0	DB	0	1	0	5	1	NA	1	0	0
95	25-avr-2013	4	2	0	77	1,62	29,3	1	83	17	NA	3	39	1	NA	36	18	21	DB	0	1	0	1	1	NA	0	0	0
96	25-avr-2013	1	2	0	#NUL!	#NUL!	#NUL!	1	80	18	NA	2	110	2	NA	0	90	39	DB	1	1	0	3	1	NA	0	0	0
97	25-avr-2013	4	1	0	115	1,65	42,2	0	55	NA	20	2	22	1	NA	9	19	1	DP	0	1		2	1		#NUL!	1	1
98	25-avr-2013	4	1	0	52	1,55	21,6	0	70	NA	16	2	22	1	NA	27	89	41	DP	0	1	1	5	1	NA	1	1	1
99	25-avr-2013	4	1	0	55	1,63	20,7	0	49	16,5	NA	2	30	1	NA	26	11	28	AC	0	1	0	5	1		0	0	0
100	25-avr-2013	1	2	0	118	1,75	38,5	1	67	19	NA	2	22	1	NA	0	0	0	DP	4	1	0	5	1		0	1	0
101	29-avr-2013	3	2	0	85	1,72	28,7	1	58	NA	19	1	73	1	NA	0	25	24	PP	0	1	1	1	1		1	1	0
102	29-avr-2013	4	1	0	91	1,65	33,4	0	80	NA	20,5	2	29	1	NA	42	28	25	PP	0	1	0	1	1		1	1	0

103	29-avr-2013	1	1	0	56	1,76	18,1	1	81	16,5	NA	1	25	1	NA	14	2	1	PP	0	1	0	1	1		0	1	0
104	30-avr-2013	1	2	0	76	1,67	27,3	0	76			1	85	1	NA	13	0	0	LCMV	0	1	3	5	0	15	1	1	1
105	30-avr-2013	2	1	0	123	1,76	39,7	0	71	20	NA	2	29	1	NA	0	3	0	LCMV	0	1	0	4	1	20	1	1	0
106	30-avr-2013	2	2	0	185	1,72	62,5	0	30	22,3	NA	1	33	1	NA	0	0	1	JFM	4	1	1	3	1	NA	0	0	0
107	30-avr-2013	1	1	0	90	1,83	26,9	0	68	17	17,5	1	108	2		25	14	0	JFM	3	1	1	3	1		0	1	0
108	14-mai-2013	3	2	0	45	1,60	17,6	1	81	14	NA	2	53	1	NA	51	13	25	LCMV	0	1	0	5	1	1	0	0	0
109	14-mai-2013	1	1	1	70	1,80	21,6	1	91	18	NA	2	65	1	NA	4	0	20	LCMV	0	1	0	5	1		1	1	0
110	13-mai-2013	1	1	0	104	1,70	36,0	1	56	NA		2	27	1	NA	9	23	14	AC	0	1	0	5	1		0	0	0
111	13-mai-2013	3	1	1	55	1,65	20,2	1	75	NA	17	1	90	1	NA	9	6	0	AC	1	0	NA	5	1		0	0	0
112	14-mai-2013	1	1	0	82	1,82	24,8	1	65	18,5	NA	2	27	1	NA	18	18	7	BL	0	1	1	5	1	NA	0	0	0
113	14-mai-2013	3	2	0	70	1,72	23,7	0	67	17	NA	1	80	1	NA	0	43	5	BL	0	1	0	1	1		0	1	0
114	14-mai-2013	3	2	0	58	1,50	25,8	0	79	15,5	NA	1	45	1	NA	33	82	0	BL	0	1	1	5	1	NA	0	0	0
115	14-mai-2013	3	1	0	97	1,78	30,6	1	78	18	NA	2	22	1	NA	0	0	16	BL	4	1	0	4	1	NA	1	0	0
116	14-mai-2013	3	1	0	82	1,57	33,3	0	55	NA	18	1	180	1	NA	96	23	100	BL	0	1	2	4	0	60	1	1	1
117	14-mai-2013	1	2	0	58	1,62	22,1	1	70	17,5	NA	2	97	1	NA	27	28	28	BL	0	1	0	1	1		0	0	0
118	14-mai-2013	3	1	0	129	1,77	41,2	1	69	21	NA	3	50	1	NA	0	0	0	BL	4	1	2	3	1	150	1	1	1
119	14-mai-2013	1	2	0	61	1,55	25,4	0	77	NA	15	2	38	1	NA	20	13	0	AC	1	1	0	5	1		0	0	0
120	14-mai-2013	1	2	0	80	1,65	29,4	1	61	NA	17,5	1	55	1	NA	4	14	5	FP	0	1	0	5	1	NA	0	1	1
121	15-mai-2013	5	1	0	65	1,68	23,0	1	62	NA	17	2	15	1	NA	63	63	21	JFM	0	1	1	1	1		1	0	0
122	15-mai-2013	5	2	1	109	1,82	32,9	1	54	NA	21	2	11	1	NA	11	1	0	JFM	4	0	NA	3	0	20	1	0	0
123	15-mai-2013	3	1	1	80	1,76	25,8	1	52	16,5	NA	2	43	1	NA	0	58	7	JFM	0	0	NA	3	1		0	1	0
124	15-mai-2013	5	1	0	124	1,88	35,1	1	46	NA	21	1	22	1	NA	0	2	0	JFM	0	1	1	5	1	30	0	1	0
125	15-mai-2013	3	2	0	77	1,58	30,8	0	44	NA	16	1	50	1	NA	0	26	2	JFM	2	1	1	5	0	25	0	0	0
126	15-mai-2013	3	2	0	98	1,67	35,1	1	57	NA	19,5	1	35	1	NA	9	4	2	JFM	4	1	0	3	1	NA	0	0	0
127	15-mai-2013	5	2	0	138	1,73	46,1	1	67	NA	21	2	27	1	NA	0	0	0	JFM	4	1	0	4	1	NA	1	1	1
128	15-mai-2013	3	1	0	94	1,54	39,6	0	74	NA	17	0	160	2	2	0	0	0	JFM	3	1	2	3	1	NA	1	1	1
129	15-mai-2013	3	1	0	105	1,68	37,2	0	39	19	NA	0	140	1	NA	0	10	0	JFM	2	1	1	2	1	NA	0	0	1
130	15-mai-2013	5	1	0	89	1,67	31,9	0	67	NA	17	1	58	1	NA	0	0	0	JFM	4	1	1	3	1	NA	0	0	0
131	15-mai-2013	3	2	0	71	1,67	25,5	1	66	NA	17	2	44	1	NA	0	0	0	JFM	0	1	1	5	1	40	0	0	0
132	15-mai-2013	3	1	0	34	1,50	15,1	0	53	NA	12,5	1	300	2	2	0	13	10	LCMV	0	1	0	1	1	40	0	0	0
133	15-mai-2013	5	2	0	64	1,52	27,7	0	70	NA	16	1	27	1	NA	0	2	2	JFM	0	1	1	1	1		1	0	0
134	15-mai-2013	5	1	1	76	1,69	26,6	1	63	17	NA	2	15	1	NA	15	22	21	JFM	0	0	NA	3	1	NA	1	1	0
135	15-mai-2013	1	2	0	97	1,58	38,9	0	59	19	NA	1	35	1	NA	39	11	32	JFM	0	1	2	3	1		1	1	0
136	15-mai-2013	1	1	0	79	1,55	32,9	1	78	18	17	1	115	2	NA	0	7	3	JFM	3	1		3	1		1	1	0
137	15-mai-2013	5	2	0	75	1,69	26,3	1	66	17	NA	2	17	1	NA	3	9	6	JFM	0	1	1	5	1		0	0	0
138	15-mai-2013	3	1	0	84	1,75	27,4	1	60	NA	17	2	22	1	NA	19	5	0	JFM	0	1	2	3	1		1	1	1
139	15-mai-2013	3	2	0	80	1,71	27,4	1	70	18	NA	3	40	1	NA	45	0	0	JFM	4	1	0	5	1		1	1	1
140	15-mai-2013	5	2	0	92	1,62	35,1	0	63	20,5	NA	1	25	1	NA	0	1	2	JFM	4	1	1	3	1	NA	0	1	0
141	15-mai-2013	1	1	0	75	1,58	30,0	0	83	18,5	18	2	80	2	NA	39	43	2	JFM	3	1	1	3	1		0	1	0
142	15-mai-2013	5	2	1	119	1,62	45,3	0	25	17,5	NA	2	40	1	NA	0	60	0	JFM	2	0	NA	5	1	NA	0	0	0
143	15-mai-2013	3	1	0	55	1,55	22,9	1	87	16	NA	3	20	1	NA	1	5	1	JFM	0	1	2	1	1		0	0	0
144	15-mai-2013	5	2	0	110	1,84	32,5	1	74	NA	21	2	55	2	3	15	9	3	JFM	3	1	2	3	1	40	1	1	0
145	15-mai-2013	1	2	0	79	1,59	31,2	0	63	NA	18	2	34	1	NA	13	23	5	JFM	0	1	1	5	1	NA	0	1	0
146	15-mai-2013	5	1	0	61	1,47	28,2	0	60	NA	16,5	2	21	1	NA	0	8	0	JFM	0	1	1	5	1	NA	1	1	0
147	15-mai-2013	1	2	1	118	1,61	45,5	0	27	17,5	NA	1	90	1	NA	8	52	1	JFM	2	0	NA	5	1	NA	0	0	0
148	15-mai-2013	5	1	0	111	1,75	36,2	1	71	NA	19	3	20	1	NA	0	38	1	JFM	0	1	1	1	1		1	1	0
149	15-mai-2013	3	2	0	75	1,70	26,0	1	69	19	NA	3	65	1	NA	0	0	0	JFM	4	1	1	2	1	20	0	0	0
150	15-mai-2013	2	2	0	83	1,71	28,4	1	48	18,5	NA	1	70	1	NA	6	48	1	JFM	0	1	1	2	1		0	0	0
151	15-mai-2013	1	1	1	125	1,77	39,9	0	32	NA	18	2	28	1	NA	30	10	0	JFM	0	0	NA	5	1	NA	0	0	0
152	15-mai-2013	3	1	0	65	1,55	27,1	0	66	15	NA	1	105	1	NA	100	56	100	JFM	0	1	1	2	1	NA	0	1	0
153	15-mai-2013	5	1	0	117	1,78	36,9	1	56	21	NA	2	11	1	NA	0	5	16	JFM	0	1	0	1	0		1	0	1
154	16-mai-2013	3	2	0	36	1,58	14,4	0	81	NA	14	1	60	1	NA	25	21	26	PP	0	1	0	1	1	NA	0	0	0
155	16-mai-2013	1	2	0	91	1,60	35,5	0	83	NA	17	1	56	1	NA	34	14	6	PP	0	1	0	5	1	NA	0	1	1
156	16-mai-2013	5	1	0	82	1,55	34,1	0	77	NA	16	1	20	1	NA	8	3	5	PP	0	1	1	2	1		0	1	0
157	16-mai-2013	1	2	0	79	1,78	24,9	1	70	17,5	NA	2	42	1	NA	2	6	0	DP	2	1	2	5	1		0	0	0
158	17-mai-2013	1	2	1	72	1,45	34,2	0	83	NA	16	2	46	1	NA	19	1	0	CV	4	0	NA	2	1	NA	0	0	0
159	17-mai-2013	1	1	0	66	1,53	28,2	0	63	NA	15	2	25	1	NA	13	52	7	CV	0	1	0	2	1	NA	0	0	0
160	16-mai-2013	5	1	1	130	1,80	40,1	1	53	21	NA	1	35	1	NA	0	0	0	DB	4	0	NA	1	0	10	1	1	1
161	16-mai-2013	3	2	0	97	1,78	30,6	1	57	NA	18	2	40	1	NA	0	2	2	DB	4	1	1	3	1	NA	0	1	1
162	16-mai-2013	5	1	0	70	1,72	23,7	1	78	17	NA	2	11	1	NA	0	2	0	DB	4	1	0	1	1	30	0	0	0
163	16-mai-2013	5	1	0	90	1,71	30,8	1	65	NA	19	2	35	1	NA	0	15	0	DB	0	1	1	3	1	NA	0	1	0
164	16-mai-2013	3	2	0	125	1,59	49,4	0	51	17	NA	1	45	1	NA	0	0	2	DB	0	1		3	1		1	1	0
165	15-mai-2013	3	1	0	66	1,74	21,8	1	77	17	NA	2	40	1	NA	0	0	0	DB	0	1	0	1	0	10	0	0	0
166	15-mai-2013	1	1	0	75	1,68	26,6	1	79	18	NA	2	40	1	NA	39	23	3	DB	0	1	1	1	1	60	1	0	0
167	02-mai-2013	5	2	0	55	1,52	23,8	0	79	NA	17	2	30	1	NA	0	0	0	LCMV	4	1	0	5	1	NA	0	1	0
168	02-mai-2013	5	1	0	86	1,72	29,1	1	80	NA	19	3	19	1	NA	0	0	0	LCMV	4	1	2	4	1	20	1	1	1
169	02-mai-2013	1	2	0	81	1,63	30,5	0	43	NA	17	1	55	1	NA	5	0	0	CV	4	1	0	3	1	NA	0	0	0
170	02-mai-2013	2	2	0	101	1,77	32,2	1	71	NA	20	2	30	1	NA	25	13	18	DP	0	1	0	5	1		1	1	0
171	02-mai-2013	2	1	0	67	1,72	22,6	1	66	18,1	NA	2	33	1	NA	11	9	8	DP	0	1		1	1		0	0	0
172	02-mai-2013	1	1	0	118	1,63	44,4	1	78	20	NA	0	15	1	NA	6	0	0	DB	0	1	0	1	1		0	0	1
173	02-mai-2013	2	2	1	87	1,82	26,3	1	34	16,9	NA	2	38	1	NA	26	5	1	DB	2	0	NA	5	1	NA	0	0	0
174	02-mai-2013	3	1	1	80	1,49	36,0	0	70	NA	16,5	2	27	1	NA	28	21	3	DB	0	0	NA	2	1	NA	0	0	0
175	02-mai-2013	1	1	0	103	1,71	35,2	1	57	NA	17,5	1	20	1	NA	83	21	7	PP	0	1	0	2	1		1	1	1
176	02-mai-2013	2	2	0	85	1,72	28,7	1	77	18,1	NA	3	42	1	NA	0	0	0	DB	0	1	1	1	1	40	0	0	1
177	06-mai-2013	2	2	0	82	1,69	28,7	1	71	NA	19	2	35	1	NA	0	0	0	BL	0	1	0	3	1		0	1	1
178	06-mai-2013	3	2	0	92	1,65	33,8	1	81	18	NA	5	56	1	NA	0	4	4	PP	0	1	0	1	1		1	1	0
179	06-mai-2013	1	1	0	87	1,82	26,3	1	82	17,5	NA	1	85	1	NA	47	3	2	AC	0	1	2	5	1	20	0	0	0
180	06-mai-2013	1	2	0	40	1,73	13,4	0	55	NA	15,5	2	40	1	NA	6	38	10	AC	0	1	0	1	0	50	0	0	0
181	10-mai-2013	1	2	1	80	1,70	27,7	0	58	NA	16	1	23	1	NA	48	0	0	HA	0	0	NA	1	1		0	0	0
182	10-mai-2013	4	2	1	71	1,64	26,4	0	79	17	NA	2	40	1	NA	22	46	0	HA	0	1	1	4	1	0	1	1	0
183	10-mai-2013	1	1	0	35	1,58	14,0	0	82	NA	14	1	13	1	NA	6	22	0	HA	0	1	1	1	0	55	0	0	0
184	18-mai-2013	1	2	0	95	1,83	28,4	1	60	18	NA	1	50	1	NA	5	3	0	DP	0	1	0	1	1	40	1	1	1
185	18-mai-2013	4	2	0	69	1,59	27,3	1	62	16	NA	2	38	1	NA	93	10	89	DP	2	1		1	1	20	0	1	0
186	19-mai-2013	4	1	0	53	1,52	22,9	0	66	16	NA	2	42	1	NA	48	31	23	DB	0	1	1	5	1	NA	0	0	0
187	18-mai-2013	5	2	0	90	1,65	33,1	1	63	NA	20	1	37	1	NA	0	0	0	LCMV	4	1	1	2	1	40	0	0	0
188	18-mai-2013	5	1	0	83	1,68	29,4	1	76	20	NA	1	45	1	NA	0	1	0	LCMV	4	1	1	2	1	35	0	0	0
189	18-mai-2013	5	1	0	112	1,63	42,2	0	51	18,5	NA	2	47	1	NA	0	31	0	HA	0	1	1	3	1	15	1	1	0
190	17-mai-2013	1	1	0	145	1,70	50,2	0	46	NA	19	2	28	1	NA	35	24	0	JFM	0	1	0	3	1	10	0	0	0
191	18-mai-2013	5	2	1	71	1,62	27,1	0	80	19	NA	1	39	1	NA	0	25	0	HA	2	0	NA	3	1	NA	1	1	0
192	18-mai-2013	1	1	0	118	1,72	39,9	1	71	17,5	NA	1	26	1	NA	31	50	48	FP	0	1	2	4	1	NA	1	1	0
193	19-mai-2013	1	1	0	111	1,53	47,4	0	59	NA	17	2	26	1	NA	28	19	7	DB	0	1	1	2	1	20	1	1	0
194	19-mai-2013	1	1	1	119	1,75	38,9	1	68	19,5	NA	2	22	1	NA	5	0	0	DB	4	0	NA	3	1	NA	0	1	0
195	23-mai-2013	4	2	0	91	1,78	28,7	1	63	18,5	NA	4	40	1	NA	4	8	10	PP	0	1	0	1	0	30	0	1	0
196	18-mai-2013	4	1	0	112	1,68	39,7	1	71	20	NA	3	25	1	NA	5	1	1	JFM	4	1	0	1	1	50	1	1	1
197	18-mai-2013	1	2	2	81	1,70	28,0	1	80	NA	18	2	26	1	NA	8	5	0	JFM	4	1	1	5	1	20	1	1	0
198	18-mai-2013	4	2	1	135	1,69	47,3	0	21	19	NA	2	45	1	NA	6	27	0	JFM	2	0	NA	5	0		0	1	0
199	18-mai-2013	4	1	0	98	1,62	37,3	0	58	NA	17	2	38	1	NA	38	3	1	JFM	1	1	2	3	1	NA	1	0	1
200	18-mai-2013	1	2	0	88	1,68	31,2	1	65	NA	18	2	24	1	NA	24	0	1	JFM	0	1	1	3	1	20	0	1	0
