# Supplementary figures and images for: A Randomized Controlled Trial on the Effect of Needle Gauge on the Pain and Anxiety Experienced during Radial Arterial Puncture
Source: PLoS One. 2015 Sep 25;10(9):e0139432. doi: 10.1371/journal.pone.0139432 (PMC4583403; doi:10.1371/journal.pone.0139432)

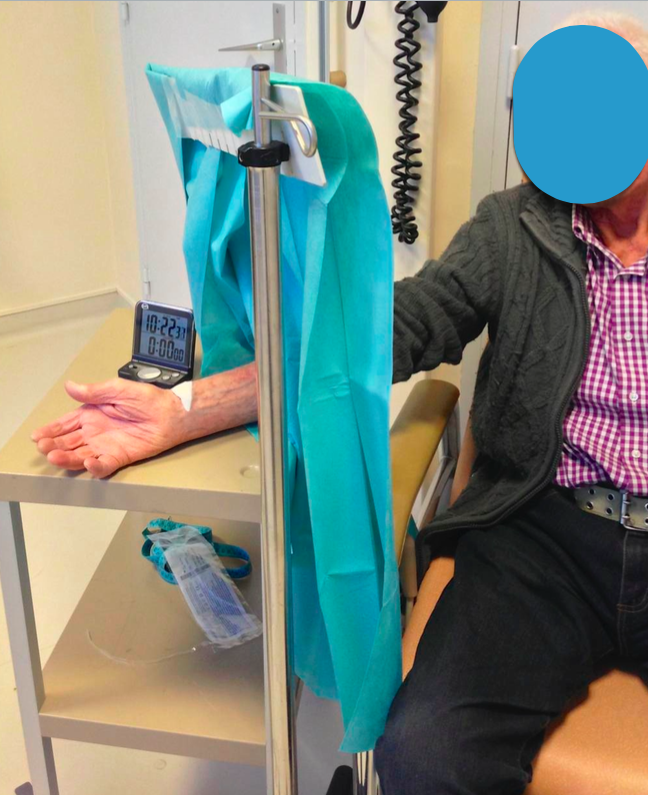

Supplement: S1 Image — Patients were installed on the right of the curtain and inserted their wrist between the curtains. Nurses were installed on the left of the curtain and performed the arterial puncture as well as the record the duration of the sampling using the chronometer. (TIFF) [file pone.0139432.s004.tiff]
